# Telomere Maintenance Is Associated with Type 2 Diabetes Remission in Response to a Long-Term Dietary Intervention without Non-Weight Loss in Patients with Coronary Heart Disease: From the CORDIOPREV Randomized Controlled Trial

**DOI:** 10.3390/antiox13010125

**Published:** 2024-01-19

**Authors:** Ana Ojeda-Rodriguez, Juan F. Alcala-Diaz, Oriol Alberto Rangel-Zuñiga, Antonio P. Arenas-de Larriva, Francisco M. Gutierrez-Mariscal, Jose D. Torres-Peña, Marina Mora-Ortiz, Juan L. Romero-Cabrera, Raul M. Luque, Jose M. Ordovas, Pablo Perez-Martinez, Javier Delgado-Lista, Elena M. Yubero-Serrano, Jose Lopez-Miranda

**Affiliations:** 1 Lipids and Atherosclerosis Unit, Internal Medicine Unit, Reina Sofia University Hospital, 14004 Cordoba, Spain; ana.ojeda@imibic.org (A.O.-R.); juanf.alcala.sspa@juntadeandalucia.es (J.F.A.-D.); oriol.rangel@imibic.org (O.A.R.-Z.); antoniop.arenas.sspa@juntadeandalucia.es (A.P.A.-d.L.); francisco.gutierrez@imibic.org (F.M.G.-M.); h42topej@uco.es (J.D.T.-P.); marina.mora@imibic.org (M.M.-O.); juanl.romero.sspa@juntadeandalucia.es (J.L.R.-C.); pabloperez@uco.es (P.P.-M.); md1delij@uco.es (J.D.-L.); elena.yubero@imibic.org (E.M.Y.-S.); 2 Department of Medical and Surgical Science, University of Cordoba, 14004 Cordoba, Spain; 3 Maimonides Biomedical Research Institute of Cordoba (IMIBIC), Av. Menendez Pidal, s/n, 14004 Cordoba, Spain; bc2luhur@uco.es; 4 CIBER Fisiopatologia de la Obesidad y Nutricion (CIBEROBN), Instituto de Salud Carlos III, 28029 Madrid, Spain; 5 Department of Cell Biology, Physiology and Immunology, University of Cordoba, 14004 Cordoba, Spain; 6 Nutrition and Genomics Laboratory, J.M. US Department of Agriculture Human Nutrition Research Center on Aging, Tufts University, Boston, MA 02111, USA; jose.ordovas@tufts.edu; 7 Instituto Madrileño de Estudios Avanzados en Alimentación (IMDEA-Food), 28049 Madrid, Spain; 8 Centro Nacional de Investigaciones Cardiovasculares Carlos III (CNIC), 28029 Madrid, Spain

**Keywords:** telomere attrition, aging, mediterranean diet, cardiovascular disease

## Abstract

In order to evaluate whether telomere maintenance is associated with type 2 diabetes remission, newly diagnosed type 2 diabetes patients without glucose-lowering treatment (183 out of 1002) from the CORDIOPREV study (NCT00924937) were randomized to consume a Mediterranean or low-fat diet. Patients were classified as Responders, those who reverted from type 2 diabetes during the 5 years of dietary intervention (*n* = 69), and Non-Responders, who did not achieve diabetes remission by the end of the follow-up period (*n* = 104). We found no differences in diabetes remission between the two diets, and we determined telomere length (TL) by measuring qPCR, telomerase activity using the TRAP assay, and direct redox balance based on the ratio of reduced glutathione (GSH) to oxidized glutathione (GSSH) via colorimetric assay. Responders exhibited higher baseline TL in comparison with Non-Responders (*p* = 0.040), and a higher TL at baseline significantly predicted a higher probability of type 2 diabetes remission (OR 2.13; 95% CI, 1.03 to 4.41). After the dietary intervention, Non-Responders showed significant telomere shortening (−0.19, 95% CI −0.32 to 0.57; *p* = 0.005). Telomere shortening was significantly pronounced in type 2 diabetes patients with a worse profile of insulin resistance and/or beta-cell functionality: high hepatic insulin resistance fasting, a high disposition index (−0.35; 95% CI, −0.54 to −0.16; *p* < 0.001), and a low disposition index (−0.25; 95% CI, −0.47 to −0.01; *p* = 0.037). In addition, changes in TL were correlated to the GSH/GSSG ratio. Responders also showed increased telomerase activity compared with baseline (*p* = 0.048), from 0.16 (95% CI, 0.08 to 0.23) to 0.28 (95% CI, 0.15 to 0.40), with a more marked increase after the dietary intervention compared with Non-Responders (+0.07; 95% CI, −0.06–0.20; *p* = 0.049). To conclude, telomere maintenance may play a key role in the molecular mechanisms underlying type 2 diabetes remission in newly diagnosed patients. However, further larger-scale prospective studies are necessary to corroborate our findings.

## 1. Introduction

Type 2 diabetes is recognized as a major public health problem. Its impact on society and healthcare is primarily attributed to the increased incidence of type 2 diabetes, which is closely linked to environmental and lifestyle factors such as sedentary lifestyle and the consumption of high-calorie diets rich in processed foods, refined sugar, or saturated fats [1]. This metabolic disease, characterized by insulin resistance and beta-cell malfunction, can result in severe micro- and macrovascular complications associated with an increased risk of cardiovascular disease [2]. 

Over the last decade, the possibility of remission has been demonstrated in type 2 diabetes, which was previously considered a progressive, irreversible disease [3,4,5]. Type 2 diabetes remission is associated with substantial weight loss via bariatric surgery [5] or dietary interventions with liquid formula caloric restriction [4] in long-duration diabetes patients. However, in the CORDIOPREV (CORonary Diet Intervention with Olive oil and cardiovascular PREVention) study population, we previously identified a set of newly diagnosed type 2 diabetes patients with coronary heart disease (CHD) who achieved diabetes remission after the long-term consumption of a healthy diet (Mediterranean or low-fat diet), without pharmacological treatment or weight loss [6,7,8]. 

Currently, research into telomeres as a potential therapeutic target seems to be a promising approach given their involvement in prediabetes and diabetes and its complications [9,10,11]. Telomeres are repetitive DNA sequences ((5′-TTAGGG-3′)_n_) bound by a protective protein complex and located at the ends of chromosomes that preserve chromosome stability and integrity. In each somatic cell division cycle, telomeres shorten because of the inability of DNA polymerase, called telomerase, to fully copy the very end of the chromosome [12]. Moreover, G-rich telomere repeat sequences are particularly prone to oxidative damage, which triggers inflammatory responses associated with cell senescence. Therefore, both telomere length (TL) and telomere shortening (a gradual reduction in TL) are considered powerful biomarkers of aging-associated pathological conditions, including heritable and acquired factors [13]. In this context, in a recent meta-analysis, 5575 patients with type 2 diabetes presented shorter TLs than 6349 healthy individuals [9], and several cross-sectional analyses have shown that TL and telomerase activity are influenced by lifestyle factors [14,15]. The susceptibility of telomeres to oxidative damage also plays a role in the development of type 2 diabetes, primarily d because of the increased oxidative stress associated with hyperglycemic conditions. Consequently, any lifestyle factors that contribute to higher oxidative stress also directly affect telomere integrity. However, few studies have evaluated the impact of lifestyle interventions on telomeric integrity in the context of a metabolic disease, where the adoption of a healthier lifestyle appears to contribute to telomere maintenance [16,17,18]. 

The primary objective of the CORDIOPREV study is to evaluate the efficacy of a Mediterranean diet as compared with a low-fat diet to prevent clinical events and mortality in patients with previous CHD through a long-term follow-up study. Here, we report the results of one secondary outcome of the CORDIOPREV study, the main objective being to evaluate whether telomere maintenance is associated with type 2 diabetes remission in response to a long-term dietary intervention without weight loss in newly diagnosed type 2 diabetes patients with CHD.

## 2. Materials and Methods

### 2.1. Study Population

The current work was conducted within the framework of the CORonary Diet Intervention with Olive oil and cardiovascular PREVention study (the CORDIOPREV study) (Clinicaltrials.gov number NCT00924937). This study is a single-center, randomized, single-blind, and controlled dietary intervention in 1002 patients with CHD randomized to follow two diets (Mediterranean diet or a low-fat diet) for seven years. The patients were recruited from November 2009 to February 2012, mostly at the Reina Sofia University Hospital (Cordoba, Spain), but other hospitals from the Cordoba and Jaen provinces (Spain) were also included. Details of the rationale and study methods, including inclusion and exclusion criteria, cardiovascular risk factors, and the patients’ baseline characteristics have been described previously [19]. Briefly, eligible patients included males and females aged 20–75 years who had established CHD, were free of clinical events related to CHD in the previous 6 months, were willing to follow a long-term monitoring study, and had no severe illnesses or an expected life expectancy lower than the length of the study. All the patients gave their written informed consent to participate in the study. The study protocol was supported by the Human Investigation Review Committee at Reina Sofia University Hospital (ref. number 1496/27/03/2009), following the institutional and good clinical practice guidelines.

**Figure 1:**
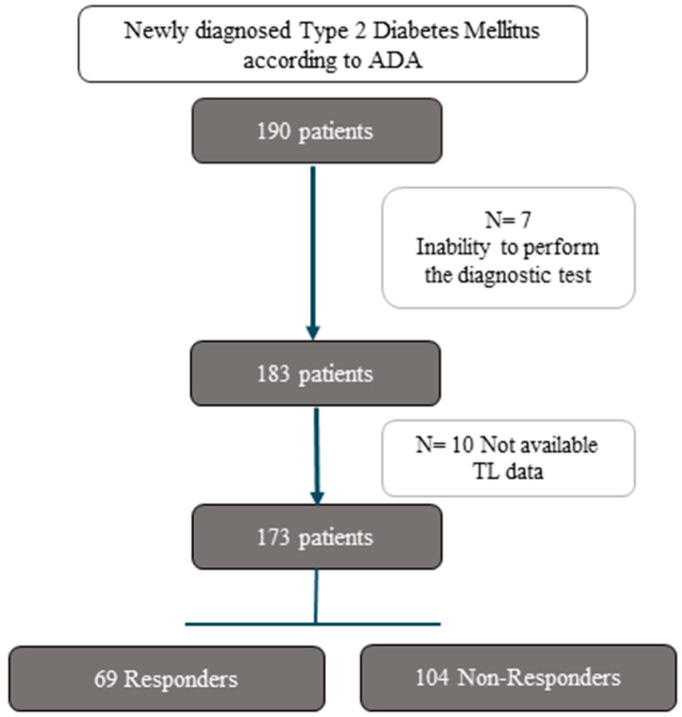
Flowchart of the study. Abbreviations: ADA, American Diabetes Association; TL, telomere length.

We included in our study, entitled the CORDIOPREV-DIRECT study, patients who were diagnosed with type 2 diabetes at the beginning of the study according to the American Diabetes Association (ADA) diagnosis criteria (fasting glucose ≥ 126 mg/dL, 2 h glucose during an oral glucose tolerance test (OGTT) ≥ 200 mg/dL, or HbA1c ≥ 6.5% (48 mmol/mol)) [20], and had not been receiving glucose-lowering treatment (190 out of 1002 patients). Following the study design, these measurements were taken at least six months after a clinical event related to CHD. Of these 190 patients, 7 were excluded because of their inability to perform the diagnostic test. Finally, a total of 183 type 2 diabetes patients were evaluated in our sub-study, of whom 73 patients reverted from type 2 diabetes during the 5 years of dietary intervention without the use of diabetes medication (Responders), while 110 did not achieve diabetes remission by the end of the follow-up period (Non-Responders). For the specific aim of this work, data on TL was available for 69 Responders and 104 Non-Responders (Figure 1). 

### 2.2. Diabetes Remission Criteria

Patients who were diagnosed with diabetes at the beginning of the study were tested yearly during follow-up and were classified as Responders or Non-Responders in the fifth year of the study. Annual follow-up testing of remission required the following criteria: (i) levels of HbA1c < 6.5% (48 mmol/mol), (ii) fasting plasma glucose < 126 mg/dL, and (iii) a 2 h plasma glucose value after 75 g in the OGTT < 200 mg dL^−1^ without the use of diabetes medication to lower blood glucose levels maintained for at least 2 years [21].

### 2.3. Dietary Intervention

The participants were randomized to follow two different healthy dietary models: (1) the Mediterranean diet, with a minimum 35% of calories from fat (22% monounsaturated, 6% polyunsaturated, and <10% saturated fatty acids), 15% proteins, and a maximum of 50% carbohydrates, and (2) a low-fat diet high in complex carbohydrates recommended by the National Cholesterol Education Program, with <30% total fat (12–14% monounsaturated, 6–8% polyunsaturated, and <10% saturated fatty acids), 15% proteins, and a minimum of 55% carbohydrates. In both diets, the cholesterol content was adjusted to <300 mg/day. Details about the diets and randomization have been previously reported and summarized [22]. In brief, the process of randomization (1:1) to the low-fat diet or the Mediterranean diet was performed by the Andalusian School of Public Health (Granada, Spain), with fixed randomization stratified in blocks, based on sex, age, and the existence of previous acute myocardial infarction. In the context of type 2 diabetes remission, we observed no differences in the percentage of Responders and Non-Responders after the two diets (Table 1). As a result, we proceeded to analyze the combined impact of the dietary intervention.

### 2.4. Laboratory Measurements

Blood samples were collected from the participants after a 12 h overnight fast at the beginning of the study and once a year during the follow-up period. Samples were collected in EDTA tubes (final concentration of 0.1% EDTA), and plasma was separated from the red cells via centrifugation at 1500× *g* for 15 min at 4 °C and immediately frozen at −80 °C. The biochemical measurements were performed at the Reina Sofia University Hospital by personnel who were unaware of the interventions. Lipid variables were assessed with a DDPPII Hitachi modular analyzer (Roche, Basel, Switzerland) using specific reagents (Boehringer-Mannheim, Mannheim, Germany). Plasma triglycerides and cholesterol concentrations were assayed via enzymatic procedures. HDL-C was measured following the precipitation of a plasma aliquot with dextran sulfate-Mg^2+^. LDL-C concentrations were calculated with the Friedewald equation using the following formula: LDL-C = CT − (HDL-C + TG/5). Glucose measurements were performed using the hexokinase method, and highly sensitive C-reactive protein levels were measured using ELISA techniques (BioCheck, Inc., Foster City, CA, USA). Reduced (GSH) and oxidized glutathione (GSSG) were determined following the methodology previously published in [23]. GSH and GSSG content were determined in the plasma samples using the BIOXYTECH^®^ GSH-400 Kit, catalog number 21011 (OXIS International Inc., Portland, OR, USA), and the GSH-412 Kit, catalog number 21040 (OXIS International Inc., Portland, OR, USA), respectively.

### 2.5. DNA Isolation from Blood Samples

Blood cells were obtained from the buffy coat fraction, which is rich in white cells such as leukocytes and mononuclear cells (neutrophils, eosinophils, basophils, lymphocytes, and monocytes). DNA isolation was carried out through the salting-out method [24] using 10 mL of Montreal–Baltimore buffer (0.32 M sucrose, 0.1 mM Tris-HCl, pH 7.5, 0.025 mM MgCl2, 1% Triton X-100) and mixing and centrifuging to separate the nuclear fraction. The nucleic pellet was homogenized with 3 mL of nuclei lysis buffer (10 mM of Tris-HCl; pH 8.2; 2 mM of EDTA; 0.4 M of NaCl) and 10% SDS and proteinase K. The DNA was precipitated with 6 M of NaCl and washed with 100% ethanol. Finally, the genomic DNA was extracted and resuspended in 500 μL of 1× TE buffer. DNA purity and concentration were evaluated via spectrophotometry using NanoDrop ND-2000 (ThermoFisher, Waltham, MA, USA).

### 2.6. Quantitative PCR Analysis of Telomere Length

TL was measured using the Cawthon method with qPCR [25]. For all samples, we estimated the relative ratio of the telomere repeat copy number (T) normalized against a single copy gene, the Homo sapiens ribosomal protein L13a gene *RPL13a* (S). The results of each PCR were relativized to a standard curve, built using a reference DNA sample. The standard curves for telomere and the *RPL13a* gene PCR consisted of eight DNA reference standards (1–25 ng). All PCRs were performed with the use of an iQ5 thermal cycler ((Bio-Rad Laboratories, Inc., Hercules, CA, USA) and a SensiFAST SYBR Lo-ROX kit (Bioline, London, UK). The coefficient of variation was 9.32% for the telomere repeat copy number and 6.76% for the single-copy gene copy number. The thermal cycler profile for both amplicons began with 95 °C incubation for 3 min to activate the polymerase, followed by 40 cycles of 95 °C for 5 s, followed by 15 s at 54 °C. The reaction mix composition was identical except for the oligonucleotide primers: 20 ng template DNA, 1× SensiFAST SYBR Lo-ROX, 200 nM reverse primer, and 200 nM forward primer. The primer sequences were (5′ → 3′):

*TeloFw*, CGGTTTGTTTGGGTTTGGGTTT GGGTTTGGGTTTGGGTT; *TeloRw*, GGCTTGCCTTACCC TTACCCTTACCCTTACCCTTACCCT; *RPL13aFw*, CCTGGAGGAGAAGAGGAAAGAGA; *RPL13aRw*, TTGAGGACCTCTGTGTATTTGTCAA. 

### 2.7. Telomerase Activity Assay

Telomerase activity was measured using the TRAP assay with the TRAP_EZE_ Telomerase Detection Kit (Intergen Co., Wellington, NZ, USA) based on an improved version of the original method described by Kim et al. [26].

### 2.8. Measurement of Insulin Resistance and Beta-Cell Function Indexes

The OGTT and the measurement of insulin resistance and beta-cell function indexes have been reported previously [6]. In brief, patients underwent a standard OGTT analyzed using the Matsuda and DeFronzo method [27] at baseline and each year during the follow-up period. After an overnight fast, blood was sampled from a vein before the oral glucose intake (0 min) and again after a 75 g flavored glucose load (Trutol 75; Custom Laboratories, Baltimore, MD, USA). Blood samples were taken at 30, 60, 90, and 120 min to determine glucose and insulin concentrations. The OGTT provided the data needed to calculate the homeostatic model assessment of insulin resistance (HOMA-IR); the insulin sensitivity index (ISI) = 10,000/√([fasting insulin (pmol/L) × fasting glucose (mmol/L)] × [mean OGTT insulin (pmol/L)] × [mean OGTT glucose (mmol/L)]); the insulinogenic index (IGI) = (30 min insulin − fasting insulin [pmol/L])/(30 min glucose − fasting glucose [mmol/L]); the hepatic insulin resistance index (Hepatic-IR_fasting_) = fasting insulin (pmol/L) × fasting glucose (mmol/L); and the disposition index (DI) = ISI × [AUC30 min insulin/AUC30 min glucose], where AUC30 min is the area under the curve between baseline and 30 min of the OGTT for insulin (pmol/L) and glucose (mmol/L) measurements, respectively, calculated using the trapezoidal method [28]. In order to assess insulin resistance (HOMA-IR, Hepatic-IR_fasting,_ and ISI indexes) and beta-cell functionality (IGI and DI indexes), we classified the patients at baseline into 4 groups according to the median of their Hepatic-IR_fasting_ and the median of their DI: (1) 44 patients who had Hepatic-IR_fasting_ below the median and DI values above the median (low Hepatic-IR_fasting_ and high DI); (2) 38 patients who had both indexes values below the median (low Hepatic-IR_fasting_ and low DI); (3) 46 patients who had Hepatic-IR_fasting_ above the median and DI values below the median (high Hepatic-IR_fasting_ and low DI); and (4) 42 patients with both indexes above the median. The second classification of our population was carried out according to the median of each index. Three patients could not be classified because of technical difficulties in acquiring the baseline DI data.

### 2.9. Statistical Analyses

The statistical analyses were performed with STATA version 14 (STATA Corp., College Station, TX, USA). We used the mean and 95% confidence interval (mean, 95% CI) for continuous variables and percentages for categorical variables. All *p*-values were 2-tailed, and a *p* < 0.05 was considered statistically significant. Student’s unpaired *t*-test was used for comparison between Responders and Non-Responders. Categorical variables were compared using chi-square tests. A logistic regression model was also run to assess the risk of type 2 diabetes remission according to TL at baseline. 

Between-group changes in TL and telomerase activity with ANCOVA were adjusted for the following variables: age, sex (males or females), BMI, waist circumference, smoking habit (never, former, or current smoker), dietary group allocation (Mediterranean diet or low-fat diet), family history of diabetes (yes or no), and pharmacological therapy (yes or no). A post hoc statistical analysis was completed using Bonferroni’s multiple comparison tests.

In previous work by our team, only DI and Hepatic-IR_fasting_ were associated with the probability of diabetes remission. Both indexes had a better predictive effect on the probability of type 2 diabetes remission [6]. For these reasons, we divided our patients into 4 groups according to the DI and Hepatic-IR_fasting_ values at baseline. We performed ANCOVA and post hoc analysis to evaluate Δchanges (defined as post-intervention minus baseline) in TL at 4 years. In addition, we classified our patients as above/below the median for each of the diabetes indexes calculated (Hepatic-IR_fasting_, HOMA-IR, DI, ISI, IGI). Next, we evaluated within- and between-group changes in TL at 4 years. 

Telomerase activity data were available for 74 participants (baseline and 4-year). We compared baseline characteristics for the participants with available data and participants without telomerase activity data using Student’s unpaired *t*-test (Supplementary Table S1). In addition, chi-square tests were performed to analyze the medication use of CHD patients, and no differences were found between groups at baseline and after the intervention (Supplementary Table S2). 

In the present work, we analyzed data at the beginning of the study and at the 4-year follow-up.

## 3. Results

### 3.1. Baseline Characteristics of the Study Population

When we compared the Responders and Non-Responders (Table 1), we observed that the Responders had a lower weight (80.4 kg; 95% CI, 77.7 to 83.0 compared with 88.8 kg; 95% CI, 85.9 to 91.6, respectively), BMI (29.8 kg/m^2^; 95% CI, 29.0 to 30.7 compared with 32.1 kg/m^2^; 95% CI, 31.2 to 33.0), waist circumference (101 cm; 95% CI, 99 to 103 compared with 108 cm; 95% CI, 106 to 110), HbA1c (6.5% compared with 6.8%), glucose (5.5 mmol/L; 95% CI, 5.3 to 5.7 compared with 6.6 mmol/L; 95% CI, 6.3 to 6.9), insulin levels (9.4 nmol/L; 95% CI, 7.7 to 11.0 compared with 13.4 mmol/L; 95% CI, 11.1 to 15.7), HOMA-IR (3.50; 95% CI, 2.36 to 4.37 compared with 4.79; 95% CI, 4.14 to 5.45), and Hepatic-IR_fasting_ (1435; 95% CI, 1083 to 1786 compared with 1951; 95% CI, 1685 to 2217) than the Non-Responders (all, *p* < 0.05). The ISI (3.14; 95% CI, 2.73 to 3.56 compared with 2.39; 95% CI, 2.15 to 2.64; *p =* 0.001) and DI (0.68; 95% CI, 0.55 to 0.81 compared with 0.43; 95% CI, 0.38 to 0.48; *p* < 0.001) were higher in Responders compared with Non-Responders. 

### 3.2. Type 2 Diabetes Remission Is Associated with Longer Telomeres 

**Figure 2:**
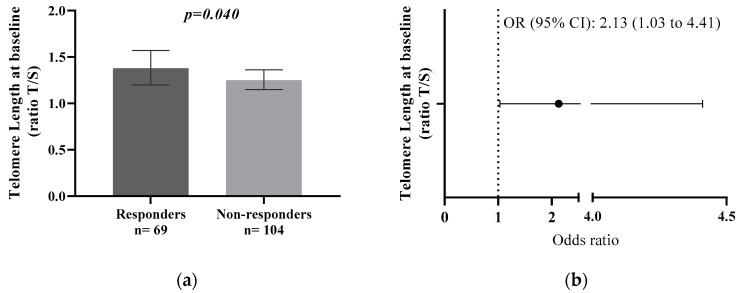
TL and type 2 diabetes remission. (**a**) TL mean at baseline in Responders and Non-Responders. (**b**) TL at baseline and risk of type 2 diabetes remission. Data are represented as the mean (95% CI). Abbreviations: CI, confidence interval; OR, odds ratio; T/S, telomere/single-copy gene. ANCOVA and logistic regression adjusted for age, sex, BMI, waist circumference, smoking habit, dietary group allocation, family history of diabetes, and pharmacological therapy.

Responders exhibited a higher baseline TL in comparison with Non-Responders (1.38; 95% CI, 1.57 to 1.20 compared with 1.25; 95% CI, 1.36 to 1.15; *p =* 0.040) (Figure 2a). We performed a logistic regression analysis to determine the probability of type 2 diabetes remission based on baseline TL. As described in Figure 2b, we found that a higher TL at baseline was associated with a higher probability of type 2 diabetes remission (OR 2.13; 95% CI, 1.03 to 4.41). 

### 3.3. Accelerated Telomere Shortening in the Non-Responders Group

**Figure 3:**
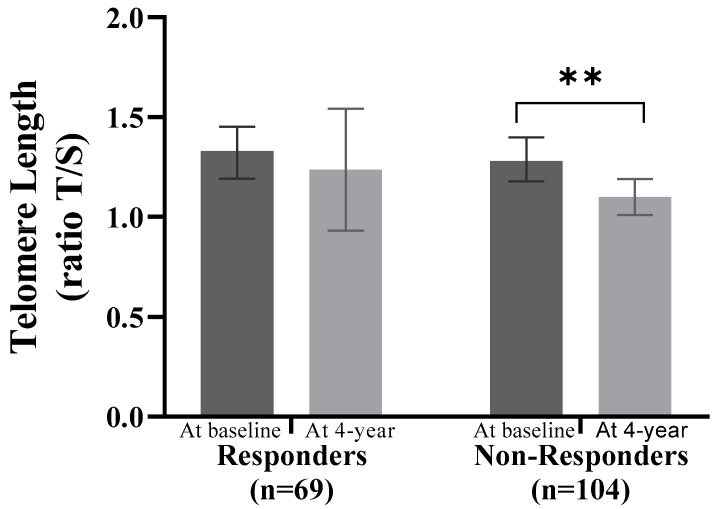
Telomere shortening in type 2 diabetes remission. Abbreviations: T/S, Telomere/single-copy gene. Data are represented as the mean (95% CI). Significant change within groups: ** *p* < 0.01. ANCOVA adjusted for age, sex, BMI, waist circumference, smoking habit, dietary group allocation, family history of diabetes, and pharmacological therapy.

We analyzed TL at baseline and at the 4-year point of the dietary intervention based on type 2 diabetes remission (Figure 3). Non-Responders showed significant telomere shortening (−0.19; 95% CI, −0.32 to 0.57; *p =* 0.005), while no significant telomere shortening was observed among Responders (−0.08; 95% CI, −0.42 to −0.06; *p =* 0.607). 

### 3.4. Telomere Shortening Is Related to Insulin Resistance 

**Figure 4:**
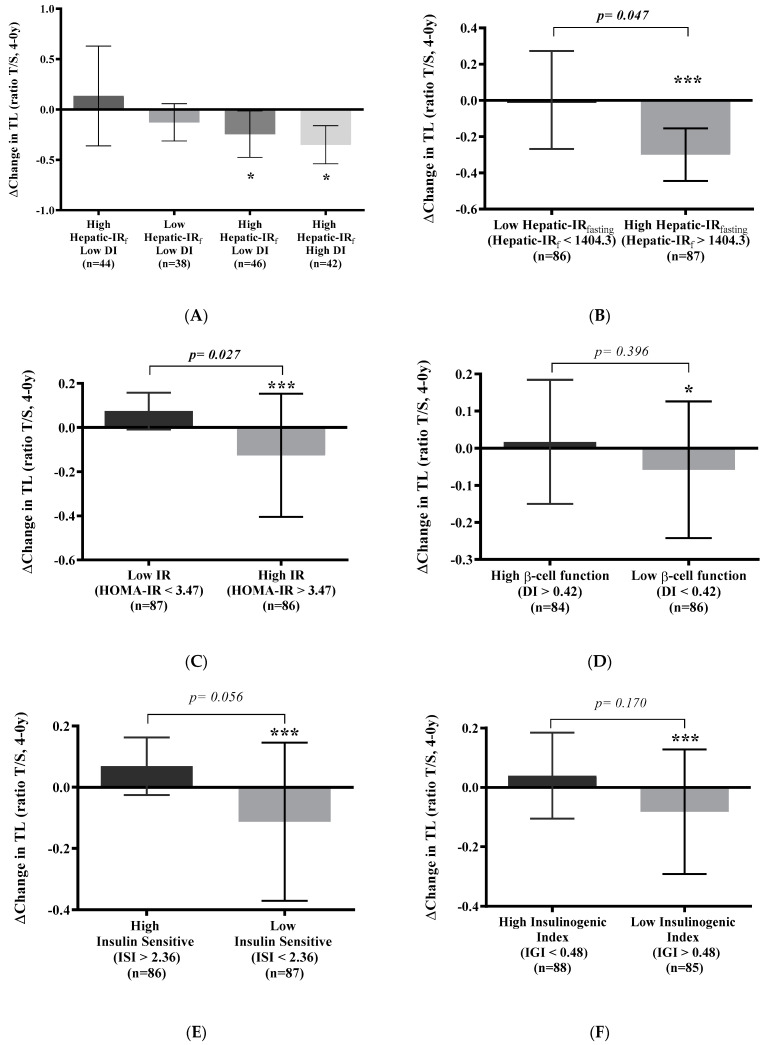
Telomere shortening based on insulin resistance and beta-cell functionality. (**A**) Telomere shortening based on a combination of hepatic insulin resistance and disposition index and based on individual indexes: (**B**) hepatic insulin resistance index derived from fasting values index, (**C**) homeostatic model assessment of insulin resistance index, (**D**) disposition index, (**E**) insulin sensitivity index, and (**F**) insulinogenic index. Data are represented as the mean (95% CI). Abbreviations: DI, disposition index; Hepatic-IRf, homeostatic model assessment of insulin resistance index; IR, insulin resistance; TL, telomere length; T/S, Telomere/single-copy gene. Significant change within groups: * *p* < 0.05, *** *p* < 0.001. ANCOVA adjusted for age, sex, BMI, waist circumference, smoking habit, dietary group allocation, family history of diabetes, and pharmacological therapy.

We evaluated Δchanges in TL (post-intervention minus baseline) according to the indexes of insulin resistance and beta-cell functionality (Figure 4) and observed that telomere shortening was significantly pronounced in type 2 diabetes patients with high Hepatic-IRfasting and high DI (−0.35; 95% CI, −0.54 to −0.16; *p* < 0.001) and low DI (−0.25; 95% CI, −0.47 to −0.01; *p* = 0.037) (Figure 4A). To assess the associations between insulin resistance, beta-cell functionality, and telomere shortening, we evaluated the Δchanges in TL (post-intervention minus baseline), looking at each of the indexes separately. Patients who presented high Hepatic-IRfasting (Figure 4B), high HOMA-IR (Figure 4C), low DI (Figure 4D), low ISI (Figure 4E), and low IGI (Figure 4F) exhibited significant telomere shortening (*p* < 0.05). Specifically, patients with high Hepatic-IRfasting (−0.15; 95% CI, −0.30 to −0.01; *p* = 0.047) and high HOMA-IR (−0.14; 95% CI, −0.30 to 0.01; *p* = 0.027) showed faster telomere shortening in comparison with patients with low values of both indexes.

### 3.5. Relationship between Change in Telomere Length and Plasma Oxidative Stress Parameter

**Figure 5:**
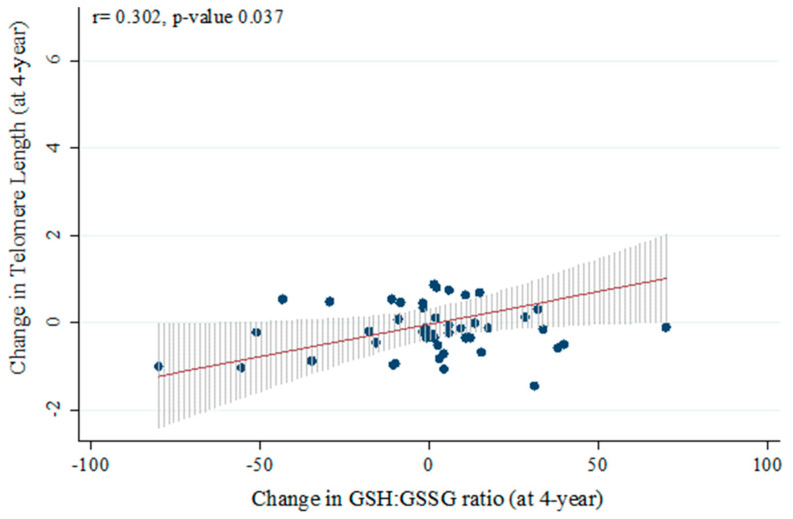
Correlation between changes in Telomere Length and changes in GSH:GSSG ratio.

To evaluate the association between telomere shortening and oxidative stress, we carried out a correlation with the ratio of reduced glutathione (GSH) and oxidized glutathione (GSSH). This correlation was evaluated in a subpopulation of the study, in which all data for GSH/GSSH and TL at baseline and year 4 of the intervention were available (*n* = 48). We found that changes in TL positively correlated with changes in the GSH:GSSG ratio (r = 0.302, *p* = 0.037. Figure 5). 

### 3.6. Increased Telomerase Activity in the Responders Group

**Figure 6:**
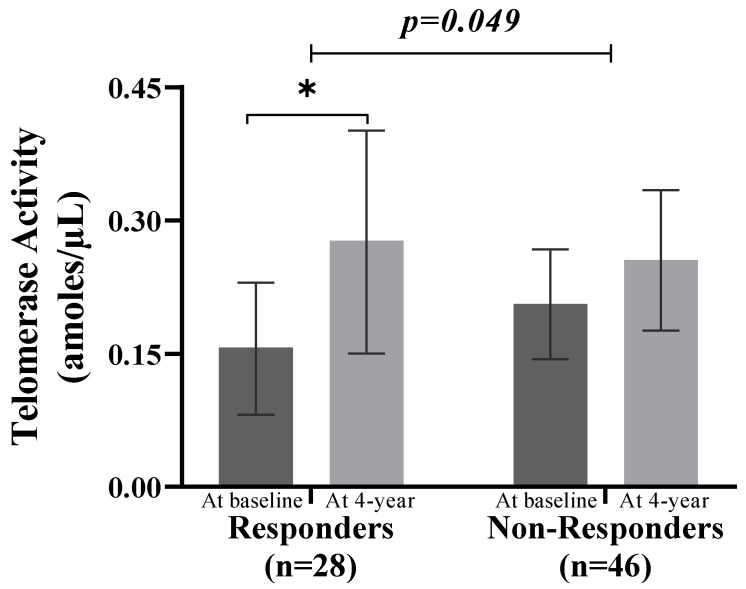
Changes in telomerase activity based on type 2 diabetes remission. Data are represented as the mean (95% CI). Significant change within groups: * *p* < 0.05. ANCOVA adjusted for age, sex, BMI, waist circumference, smoking habit, dietary group allocation, family history of diabetes, and pharmacological therapy.

Telomerase activity data were available for 74 participants (at baseline and after 4 years). The baseline characteristics for these 74 participants did not differ significantly from the characteristics of those without telomerase data (Supplementary Table S1). No differences were found in baseline telomerase activity between the two groups (0.21; 95% CI, 0.14 to 0.27 for Non-Responders compared with 0.16; 95% CI, 0.08 to 0.23 for Responders, *p =* 0.322) (Figure 6). Responders showed increased telomerase activity after dietary intervention compared with baseline (*p* = 0.048), from 0.16 (95% CI, 0.08 to 0.23) to 0.28 (95% CI, 0.15 to 0.40). In addition, we found that Responders showed increased telomerase activity (difference between Responders and Non-Responders: +0.07; 95% CI, −0.06 to 0.20; *p =* 0.049) in comparison with Non-Responders after dietary intervention (Figure 6). 

## 4. Discussion

Our study presents a comprehensive overview of the involvement of telomere maintenance in the molecular mechanisms underlying diabetes remission in newly diagnosed type 2 diabetes patients with CHD. Indeed, we have shown that patients who did not achieve diabetes remission (Non-Responders) after 5 years of dietary intervention exhibited shorter telomeres at baseline, with the length of telomeres having a predictive role in type 2 diabetes remission. We also identified significant telomere shortening in Non-Responders, which was related to a worse profile of insulin resistance and beta-cell functionality. Telomerase activity was significantly increased in patients who remitted from type 2 diabetes (Responders) in comparison with Non-Responders. 

Recent studies suggest the possibility of remission in type 2 diabetes through weight loss via caloric restriction interventions (4). Among patients in the DiRECT trial (patients characterized by an average diabetes duration of 3 years, higher obesity levels, and previous use of anti-diabetic medication), remission was strongly correlated with the degree of weight loss (4). In contrast to this noteworthy finding in patients with established diabetes, within the CORDIOPREV study population, we have identified a set of newly diagnosed type 2 diabetes patients who achieved diabetes remission without weight loss or anti-diabetic medication. In the early stage of type 2 diabetes, the probability of diabetes remission is higher even in those cases where there was no substantial weight loss, and a lifestyle intervention appeared to be useful to diabetes remission. Our study sheds light on one of the possible mechanisms involved in the remission of type 2 diabetes following a dietary intervention without caloric restriction, such as telomere maintenance, and its association with beta-cell functionality and insulin resistance.

To the best of our knowledge, this is the first study to evaluate the involvement of telomere maintenance in diabetes remission and, in particular, in the context of secondary cardiovascular prevention. Previous studies have consistently demonstrated the presence of shorter TLs in patients with type 2 diabetes compared with healthy subjects [9]. In line with this, evidence points to the fact that patients with newly diagnosed diabetes, like the patients in our study, also exhibit significantly shorter TLs than control subjects without type 2 diabetes [29]. Furthermore, in patients with newly diagnosed diabetes, those with better plasma glucose (HbA1c < 7%) had longer TLs, suggesting that TL could reflect plasma glucose status [30]. In our study, a shorter TL was also associated with a lower probability of type 2 diabetes remission, which is consistent with prospective studies reporting up to a 2.0-fold increased risk of developing type 2 diabetes in healthy subjects [31] and a 3.5-fold higher risk of all-cause mortality in type 2 diabetes patients with shorter TLs [32]. We also suggest that TL may also have a predictive role in the remission of type 2 diabetes, as it does in its development. 

In our study, we found faster TL shortening in Non-Responders, which was associated with higher insulin resistance and lower beta-cell functionality. In patients with prediabetes, two randomized clinical dietary intervention trials have specifically evaluated telomere maintenance. Hovatta et al. observed that TL increased in two-thirds of the patients, a result inversely associated with type 2 diabetes development after dietary intervention [33]. In contrast, Canudas et al. reported no significant changes in TL after the long-term consumption of pistachios, although the changes were negatively correlated with HOMA-IR [34]. In line with these results, when we classified the patients separately according to the insulin resistance and beta-cell functionality indexes, we observed notable telomere shortening in patients with higher insulin resistance status (high Hepatic-IR_fasting_ and high HOMA-IR). In general, telomere maintenance can be explained by the mechanism involved in reducing biological stress (inflammation or oxidative stress) and telomerase activation [35]. In this context, while certain mechanisms have been described in type 2 diabetes development, the relationship between telomere maintenance and diabetes remission remains unstudied. High glucose levels and increased oxidative stress associated with insulin resistance might interfere with telomerase activity or have a direct effect on the oxidation of G-rich telomeric strands, resulting in shortened telomeres. This scenario, in turn, could lead to senescence in pancreatic beta-cells; reduced beta-cell mass; impaired glucose tolerance; and, subsequently, diabetes development [36]. Here, our results suggest that the possible mechanism involved in such accelerated telomere shortening is insulin resistance rather than beta-cell functionality, supported by the significant differences observed between the groups categorized on the basis of their insulin resistance status. In addition, accelerated telomere attrition in the group with higher Hepatic-IR may be associated with high levels of intrahepatic triglycerides, which is a characteristic of high insulin resistance status. In this area, several studies have established an association between a shorter TL and nonalcoholic fatty liver, which is one of the main metabolic conditions related to Hepatic-IR in the context of type 2 diabetes [37]. In this sense, our study showed that changes in TL are positively associated with changes in the GHS:GSSG ratio, one of the most used markers of oxidative damage. A number of original studies, such as the Framingham Heart Study [38] and the Bogalusa Heart Study [39], have illustrated this connection between telomere shortening, insulin resistance, and oxidative stress.

On the other hand, we also observed an increase in telomerase activity in Responders in comparison with Non-Responders. Under certain conditions, it is theoretically possible that telomerase activity, in normal stem cells, may slow down the rate of telomere shortening. In patients with type 2 diabetes, poor glycemic control is associated with decreased levels and telomerase activity, which increases susceptibility to cardiovascular disease [40,41]. Over the last few years, a significant increase in telomerase activity has been reported after nutritional interventions (e.g., n-3, vitamin D, or Q10 supplementation), which suggests the protective or detrimental effect of the diet on telomere maintenance [42]. 

## 5. Conclusions

No study has yet evaluated the influence of the telomere–telomerase system on patients with type 2 diabetes when blood glucose levels are controlled. Here, we postulate that the molecular mechanisms involved in the development of diabetes, including accelerated telomere shortening and reduced telomerase activity, could plausibly underlie the observed metabolic flexibility found in type 2 diabetes remission. 

One major strength of our study is the approach used to assess type 2 diabetes remission after dietary intervention with two healthy diets, without caloric restriction or weight loss, and without any pharmacological treatment in patients with CHD. Our results open up new avenues related to the study of telomere maintenance in type 2 diabetes remission, which is of major importance since the progression of type 2 diabetes in these patients severely increases the risk of new cardiovascular events. Nevertheless, the results of the present study should be interpreted in the context of its limitations. Firstly, this research is based on a long-term, well-controlled dietary intervention, which ensures the quality of the study but may not reflect the level of compliance in a free-living population. Secondly, type 2 diabetes was not the primary endpoint of the CORDIOPREV trial but rather a secondary objective of this study, which means it is not possible to assess causality from our observations. Thirdly, although CORDIOPREV is a randomized controlled trial, we diagnosed 190 patients with diabetes type 2 at baseline and classified them as Responders or Non-Responders after dietary intervention, without detecting any significant differences based on the dietary model. This classification in the fifth year of the study was an essential step in conducting an observational analysis of the association between telomere maintenance and diabetes remission. Finally, our study is limited by a small sample size, consisting of CHD patients, which prevents the generalization of the findings to other populations. Further molecular and larger-scale prospective studies are needed to offer a more comprehensive explanation that links all the results shown in our study.

In conclusion, our study suggests that telomere maintenance could be associated with the remission of type 2 diabetes following two healthy dietary interventions without caloric restriction in newly diagnosed patients with CHD. Additionally, this study highlights the potential of targeting telomerase activity as a therapeutic strategy for type 2 diabetes management. In future studies, we hope to reveal new mechanisms in telomere maintenance and their relative importance in type 2 diabetes remission, with the goal of providing more precise, personalized medical care by tailoring the treatments to the circumstances of each patient.

## Figures and Tables

**Table 1:** Baseline characteristics of the study population.

	Responders (*n* = 69)	Non-Responders (*n* = 104)
Males/Females (*n*)	58/11	86/18
Low-fat diet/Mediterranean diet (*n*)	37/32	60/44
Age (years)	60 (58 to 62)	59 (57 to 61)
Height (m)	1.64 (1.62 to 1.66)	1.66 (1.65 to 1.67)
Weight (kg)	80.4 (77.7 to 83.0)	88.8 (85.9 to 91.6)
Body mass index (kg/m^2^)	29.8 (29.0 to 30.7)	32.1 (31.2 to 33.0)
Waist circumference (cm)	101 (99 to 103)	108 (106 to 110)
HDL-cholesterol (mmol/L)	43 (40 to 46)	41 (39 to 43)
LDL-cholesterol (mmol/L)	88 (82 to 93)	94 (89 to 100)
C-reactive protein (nmol/L)	2.75 (2.29 to 3.22)	2.95 (2.51 to 3.40)
Triglycerides (mmol/L)	133 (114 to 152)	149 (136 to 162)
HbA1c (%; mmol/mol)	6.53 (6.36 to 6.71); 48	6.82 (6.65 to 6.98); 51
Glucose (mmol/L)	5.5 (5.3 to 5.7)	6.6 (6.3 to 6.9)
Insulin (nmol/L)	9.4 (7.7 to 11.0)	13.4 (11.1 to 15.7)
HOMA-IR	3.50 (2.36 to 4.37)	4.79 (4.14 to 5.45)
ISI	3.14 (2.73 to 3.56)	2.39 (2.15 to 2.64)
IGI	0.70 (0.30 to 1.10)	0.68 (0.40 to 0.97)
Hepatic-IRfasting	1435 (1083 to 1786)	1951 (1685 to 2217)
DI	0.68 (0.55 to 0.81)	0.43 (0.38 to 0.48)

Values expressed as mean (95% CI). Frequencies in categorical variables. Responders group: patients who reverted from type 2 diabetes after 60 months of dietary intervention follow-up. Non-Responders group: patients who remained type 2 diabetic after 60 months of follow-up. Abbreviations: DI, disposition index; Hepatic-IRfasting, hepatic insulin resistance index derived from fasting values; HOMA-IR, homeostatic model assessment of insulin resistance; IGI, insulinogenic index; ISI, insulin sensitivity index.

## Data Availability

The data presented in this study are available on request from the corresponding author. Collaborations with the CORDIOPREV study are open to biomedical institutions, always after an accepted proposal for a scientific work. Depending on the nature of the collaboration, electronic data, hard copy data, or biological samples should be provided. All collaborations will be made after a collaboration agreement. The terms of the collaboration agreement will be specific for each collaboration, and the extent of the shared documentation (i.e., de-identified participant data, data dictionary, biological samples, hard copy, or other specified data sets) will also be specifically set in light of each work.

## Supplementary Materials

The following supporting information can be downloaded at https://www.mdpi.com/article/10.3390/antiox13010125/s1: Table S1: Comparison of study population versus population excluded for missing telomerase activity; Table S2. Patients’ most frequent use of medications at baseline and after the intervention.
