# Supplementary material for: Telomere Maintenance Is Associated with Type 2 Diabetes Remission in Response to a Long-Term Dietary Intervention without Non-Weight Loss in Patients with Coronary Heart Disease: From the CORDIOPREV Randomized Controlled Trial
Source: Antioxidants (Basel). 2024 Jan 19;13(1):125. doi: 10.3390/antiox13010125 (PMC10813241; doi:10.3390/antiox13010125)
Supplement: Supplementary file 1 [file antioxidants-13-00125-s001.zip › Supplementary Table S1.pdf]

Supplementary Table S1. Comparison of Study Population versus Population Excluded for Missing Telomerase Activity.

	**Population with telomerase activity data (n= 74)**	**Population without telomerase activity data (n= 109)**
Responders/Non-Responders	28/46	45/64
Males/Females (n)	63/11	89/20
Low-Fat diet/Mediterranean diet	42/32	61/48
Age (years)	59 (57-61)	61 (59-63)
Telomere Length (ratio T/S)	1.29 (1.11-1.47)	1.31 (1.20-1.43)
Height (m)	1.66 (1.64-1.67)	1.65 (1.63-1.66)
Weight (kg)	84.6 (81.9-87.3)	85.6 (82.7-88.5)
Body mass index (kg/m^2)	30.8 (29.9-31.8)	31.4 (30.6-32.3)
Waist circumference (cm)	105 (103-107)	106 (104-108)
HDL-cholesterol (mmol/L)	41 (39-44)	42 (40-44)
LDL-cholesterol (mmol/L)	89 (83-95)	10 (89-99)
Triglycerides (mmol/L)	140 (124-155)	144 (130-158)
C-reactive protein (nmol/L)	3.49 (2.50-4.49)	3.78 (2.97-4.60)
HbA1c (%)	6.67 (6.49-6.86)	6.70 (6.54-6.85)
Glucose (mmol/L)	111.9 (105.5-118.4)	109.7 (105.7-113.7)
Insulin (nmol/L)	11.6 (10.0-13.2)	11.9 (9.6-14.2)
HOMA-IR	4.19 (3.33-5.06)	4.38 (3.75-5.03)
ISI	2.76 (2.40-3.130)	2.62 (2.35-2.90)
IGI	0.51 (0.27-0.77)	0.79 (0.47-1.13)
Hepatic-IR$_{fasting}$	1723 (1376-2070)	1770 (1514-2026)
DI	0.55 (0.43-0.67)	0.51 (0.46-0.57)

Values expressed as mean (95%CI). Frequencies in categorical variables. Abbreviations: DI, Disposition Index; Hepatic-IR$_{fasting}$, Hepatic Insulin Resistance Index derived from Fasting values; IGI, InsulinoGenic Index; ISI, Insulin Sensitivity Index.
