# Supplementary material for: Telomere Maintenance Is Associated with Type 2 Diabetes Remission in Response to a Long-Term Dietary Intervention without Non-Weight Loss in Patients with Coronary Heart Disease: From the CORDIOPREV Randomized Controlled Trial
Source: Antioxidants (Basel). 2024 Jan 19;13(1):125. doi: 10.3390/antiox13010125 (PMC10813241; doi:10.3390/antiox13010125)
Supplement: Supplementary file 1 [file antioxidants-13-00125-s001.zip › Supplementary Table S2.pdf]

Supplementary Table S2. Patients' most frequent use of medications at baseline and after the intervention.

	Baseline medication (%)			After Intervention medication (%)		
	**Responders N=69**	**Non Responders N=104**	***p-value***	**Responders N=69**	**Non Responders N=104**	***p-value***
Statins	86	88	0.705	98	98	0.845
Beta-blockers	79	81	0.369	77	79	0.688
Anti-aggregates	97	99	0.339	94	97	0.412
Proton pump inhibitors	89	86	0.550	79	80	0.882

Values expresses as percentage (%). Chi-square test.
